# Biases in Understanding Attention Deficit Hyperactivity Disorder and Autism Spectrum Disorder in Japan

**DOI:** 10.3389/fpsyg.2018.00244

**Published:** 2018-02-28

**Authors:** Mami Miyasaka, Shogo Kajimura, Michio Nomura

**Affiliations:** ^1^Department of Cognitive Psychology in Education, Graduate School of Education, Kyoto University, Kyoto, Japan; ^2^Japan Society for the Promotion of Science, Tokyo, Japan; ^3^Brain Science Institute, Tamagawa University, Tokyo, Japan

**Keywords:** attention deficit hyperactivity disorder, autism spectrum disorder, assessment, clinical decision making, misdiagnosis, bias

## Abstract

Recent research has shown high rates of comorbidity between attention deficit hyperactivity disorder (ADHD) and autism spectrum disorder (ASD) and difficulties regarding differential diagnosis. Unlike those in Western countries, the Japanese ADHD prevalence rate is lower relative to that of ASD. This inconsistency could have occurred because of cultural diversities among professionals such as physicians. However, little is known about attitudes toward ADHD and ASD in non-Western cultural contexts. We conducted two experiments to identify biases in ASD and ADHD assessment. In Study 1, we examined attitudes toward these disorders in medical doctors and mental health professionals, using a web-based questionnaire. In Study 2, medical doctors and clinical psychologists assessed four fictional cases based on criteria for ADHD, ASD, oppositional defiant disorder, and disinhibited social engagement disorder (DSED). Diagnosis of ASD was considered more difficult relative to that of ADHD. Most participants assessed the fictional DSED case as ASD, rather than DSED or ADHD. The results provide evidence that Japanese professionals are more likely to attribute children’s behavioral problems to ASD, relative to other disorders. Therefore, Japanese therapists could be more sensitive to and likely to diagnose ASD, relative to therapists in other countries. These findings suggest that cultural biases could influence clinicians’ diagnosis of ADHD and ASD.

## Introduction

The behavioral, mood, and biological characteristics of mental disorders often overlap and occur comorbidly. Attention deficit hyperactivity disorder (ADHD) and autism spectrum disorder (ASD) are the most difficult developmental disorders to distinguish from each other (American Psychiatric Association [APA], 2013). ADHD is a neurodevelopmental disorder characterized by inattention, hyperactivity, and impulsivity, and one or more symptoms often manifest during childhood (American Psychiatric Association [APA], 2013). The prevalence of ADHD is between 5.0 and 7.2% in children (Willcutt, 2012; American Psychiatric Association [APA], 2013; Thomas et al., 2015). ASD is also a neurodevelopmental disorder, with a prevalence of approximately 1.0%, and it is characterized by persistent deficits in social communication and interaction across multiple contexts, and restricted, repetitive patterns of behavior, interests, or activity (American Psychiatric Association [APA], 2013).

Although the main characteristics of ADHD and ASD differ, the two disorders overlap partially. For example, high rates of ADHD and ASD comorbidity have been reported (Yoshida and Uchiyama, 2004), and individuals with ADHD often exhibit interpersonal communication issues similar to those in observed in individuals with ASD (Nijmeijer et al., 2009). In addition, people with ASD sometimes present with impulsivity and inattention, which are characteristics of ADHD (Nicolson and Castellanos, 2000). Moreover, neuroimaging studies have shown that the two disorders share abnormalities in the inferior parietal lobe (Brieber et al., 2007) and precuneus (Di Martino et al., 2013). Although diagnostic agreement between physicians and psychotherapists is important because treatments differ between ADHD and ASD (Perry, 1998), psychological assessment is difficult, and several studies have indicated that inaccurate diagnoses constitute a serious problem (Mayou and Hawton, 1986; Ruggero et al., 2010; Matson and Kozlowski, 2011). Further, because ADHD and ASD symptoms overlap, detection and differential diagnosis are difficult for these disorders. For example, a study conducted by Miodovnik et al. (2015), with a study sample that included predominantly (i.e., 80–90%) White or Black children, found that 20% of those diagnosed with ASD had initially been diagnosed with ADHD. Therefore, the researchers posited that an incorrect diagnosis of ADHD could delay the diagnosis of ASD.

Interestingly, the prevalence of ADHD and ASD provided in the Diagnostic and Statistical Manual of Mental Disorders, Fifth Edition (DSM-5; American Psychiatric Association [APA], 2013) are inconsistent with those reported by the National Institute of Special Needs Education, Japan (NISE). The National Institute of Special Needs Education Japan [NISE] (2015) reported that the number of students with ASD who required special needs education was higher than the number of students with ADHD. In addition, 42.4% of the 174,881 elementary or junior high students who enrolled in classes for special needs education registered based on ASD or emotional disturbances. Moreover, 12,308 students with ASD received special needs services in resource rooms, which provide education to children with special needs in a regular classroom. In contrast, no special needs education classes were provided for students with ADHD, and 10,324 of these students received special needs services in resource rooms. Furthermore, Getahun et al. (2013) showed a possible cultural difference in increasing tendencies to diagnose ADHD, suggesting that the number of Asian/Pacific Island children diagnosed with ADHD remained unchanged over time (approximately 1 per 100 children), while the numbers of Black, Hispanic, and White children diagnosed with ADHD increased significantly between 2001 and 2010. The reason for the high ASD prevalence in Japan could be that children with ADHD who also met the criteria for ASD were diagnosed with ASD, rather than ADHD, before the DSM-5 was published. However, this inconsistency is questionable, because these diagnostic criteria are the same internationally. One possible explanation for this finding is that physicians’ or psychotherapists’ cultural backgrounds underlie the higher ASD rates and lower ADHD rates. Some researchers have attempted to elucidate biomarkers for ADHD, as the use of the subjective approach involves risk of under- or over-diagnosis of ADHD symptoms (e.g., Zhu et al., 2008; Monden et al., 2015). Therefore, consideration of the aforementioned inconsistencies should include subjectivity in diagnosis and factors that affect subjectivity, such as cultural differences.

Daley (2002) noted that illness awareness and concepts are affected by what behavior is noticed first and whether it is perceived as problematic. Furthermore, as Ravindran and Myers (2012) noted in their review, culture plays an important and distinct role in perceptions of health, illness, and disability; for example, there are varied and widespread perceptions of disability among cultures and the definition of a “handicap” depends on cultural context. Mann et al. (1992) provide a key example: They asked mental health professionals from four countries to examine 8-year-old boys’ behavior using videotaped vignettes and found that Chinese and Indonesian clinicians gave significantly higher scores for hyperactive-disruptive behaviors than did Japanese and American clinicians. They suggested that this result reflected a difference in cultural standards for appropriate child behaviors; for example, emotional control and conformity in preschool in China, permissiveness and student self-regulation with little direct teacher discipline during lower school grades in Japan, and individual expression and creativity in United States. These observations led to the idea that, although ADHD diagnoses have become more globalized and the attitude, diagnoses, and treatment have changed in the past decades (Conrad and Bergey, 2014), cultural differences still affect assessment (Mason et al., 2014).

Studies examining developmental disorders have been conducted mainly in Western cultures (Sun and Allison, 2010). However, prevalence rates are affected by the circumstances present in each country. For example, the ASD prevalence in China (i.e., approximately 10 per 10,000 population) is lower relative to those in Western countries (Sun and Allison, 2010). One of the reasons for this difference could be that developmental disorders are not widely understood in China, as preschool teachers’ lack of knowledge regarding ASD (Liu et al., 2016) is problematic. In contrast, levels of familiarity with developmental disorders are high in Japan (Takahara and Tsuda, 2012). Therefore, other factors could explain why the ASD rate is higher, relative to the ADHD rate, in non-Western cultural contexts where developmental disorders are well known, such as Japan.

Heuristics constitute a key factor affecting clinical judgment, as they play a vital role in the reasoning process (Vázquez-Costa and Costa-Alcaraz, 2013). Humans cannot process all the information that they are exposed to because time and cognitive resources are limited. Heuristics allow for rapid decision-making, but could also lead to cognitive error in clinical judgment. For example, “premature closure” refers to the tendency to accept a potential diagnosis and end the decision-making process prematurely; social and cultural contexts could influence this mental shortcut. Takahara and Tsuda (2012) examined familiarity with terms related to developmental disorders in the general population, with responses provided using four-point Likert scales (1 = *I do not know the term*, 2 = *I have heard the term*, 3 = *I know something about the term*, and 4 = *I know the term well*). According to the results, the proportion of people who were familiar with ADHD was higher than 20% (including *I know something about the term* and *I know the term well*), while over 60% of people were familiar with ASD (however, rates for Asperger’s syndrome, high-functioning autism, and pervasive developmental disorder were low). The most general sources of information included newspapers and television, indicating that there were numerous opportunities to obtain information concerning ASD in everyday life. Furthermore, Asian people characteristically attach high value to non-verbal information (Hall, 1976; Ishii et al., 2003), and difficulty with non-verbal skills is one of the main characteristics of ASD. Fundamental research has shown that a high degree of familiarity with stimuli affects psychological processes, including those involving perception or cognitive function such as rapid visual search or low-threshold perception (Howes and Solomon, 1951; Wang et al., 1994). Therefore, it is possible that Japanese people adopt information regarding highly familiar ASD-related characteristics.

Based on these findings, the amount of attention paid to ASD could be greater relative to that paid to ADHD in Japan, which is contrary to tendencies observed in the West. However, little is known regarding attitudes toward ADHD and ASD in non-Western cultural contexts. Characterizing how ADHD and ASD are recognized could enhance current understanding and identify factors leading to inappropriate diagnosis and assessment. This could help to identify future directions in the development of psychology assessment methods suitable for use with people of diverse ethnicities. The objective of this study was to examine the characteristics of the assessment attitude toward ADHD among Japanese clinical professionals and to discuss them in a cultural context. The aim of Study 1 was to examine participants’ recognition of ADHD, relative to that of ASD. The aim of Study 2 was to determine whether clinicians’ biases were reflected in the assessment of fictional cases, based on the results of Study 1.

## Study 1

### Survey Examining Similarities and Differences Between Attitudes Toward ADHD and ASD

In Study 1, we sought to determine whether ADHD and ASD were regarded as distinct disorders and explored participants’ understanding of the similarities and differences between them. Because of the overlap between ADHD and ASD symptoms, we expected some participants to regard ADHD and ASD as a single disorder. In addition, we examined participants’ perspectives on the relationship between the two disorders. Our hypothesis was that participants would focus on non-verbal communication skills and brain activity in relation to psychological and biological factors, respectively. The participants were professionals providing developmental support (e.g., medical doctors and psychotherapists).

### Method

#### Participants

We used Qualtrics.com to conduct a web survey. Written requests for responses were sent to 516 specialized agencies in the developmental support field in Japan (i.e., hospitals, elementary schools, child consultation centers, and a support center for developmental disorders). Either 10 or 11 organizations were randomly selected from each prefecture. The agencies were informed of the purpose of the study, voluntary nature of participation, and anonymity of the data collected. Therefore, each agency informed its constituents voluntarily. Participants were provided with similar explanations. The study was conducted from the beginning of March to the end of April 2014. Thirty-four adults participated in Study 1 (17 women; mean age [*M*_age_] = 43.03 ± 13.89 years). The participants did not receive compensation.

#### Questionnaire Content

We measured participants’ attitudes to ADHD and ASD (i.e., understanding regarding psychological and biological factors and conceptual understanding), the numbers of diagnoses, and knowledge accuracy. Terms were based on the Diagnostic and Statistical Manual of Mental Disorders, Fourth Edition, Text Revision (DSM-IV-TR; American Psychiatric Association [APA], 2000) in the study (i.e., pervasive developmental disorders instead of ASD), even though this was not the most recent revision, because we believed that the participants would be more familiar with the DSM-IV-TR than the DSM-5 (American Psychiatric Association [APA], 2013).

#### Psychological and Biological Factors

Participants were asked to provide free descriptions of the similarities and differences between the psychological and biological factors of ADHD and ASD.

#### Conceptual Understanding

Participants chose the statement that described their own understanding most accurately from the following four statements regarding attitudes toward the relationship between ADHD and ASD, and provided a reason for the choice (we explained that there were no incorrect answers): “I consider ADHD and ASD different disorders” (*different*); “even though ADHD and ASD are different disorders, it is not necessary to distinguish between them diagnostically” (*differentiation unnecessary*); “I believe that ADHD and ASD are the same disorder” (*same*); and “I do not consider ADHD and ASD disorders” (*not disorders*). The “*differentiation unnecessary*” and “*same*” statements were slightly similar. We assumed that participants who selected the former believed that ADHD and ASD were two distinct disorders, while those who chose the latter regarded ADHD and ASD as a single disorder.

#### Knowledge Accuracy

We measured the accuracy of participants’ knowledge regarding developmental disorders, as it could be related to attitude bias. The questionnaire consisted of 16 true–false questions regarding developmental disorders (e.g., “ADHD always includes these three traits: inattention, hyperactivity, and impulsivity”; the correct response is “false”), which were adapted from those used by Kikuchi (2011), and partly reworded.

#### Diagnoses

Participants reported the numbers of clients for whom they had provided treatment for each of the following diagnoses: ADHD, ASD, and comorbid ADHD and ASD (ADHD + ASD).

#### Compliance With Ethical Standards

All study participants provided informed consent; ethical approval was not required for this study in accordance with the national and institutional guidelines.

#### Data Analyses

Statistical analyses were performed using R3.4.0 for Mac OS (R Core Team, 2017) and statistical power was calculated using G^∗^Power 3.1 (Faul et al., 2009). We analyzed the diagnosis rate for each category of disorders. We calculated the proportion of clients with each of the three diagnoses treated by each participant (i.e., the total for the three diagnoses was 100% for each participant). A one-way analysis of variance (ANOVA) was performed following angular transformation.

Participants’ responses were classified according to the items concerning conceptual understanding. We performed a chi-square test using the frequencies for the four opinions to determine whether participants’ understanding was consistent with operational diagnostic criteria. Thereafter, participants’ reasons for their choices were classified into categories according to similar content, and each category was summarized.

Similarly, responses concerning psychological and biological similarities and differences were classified according to similar content, and each category was summarized. We found several multiple responses regarding psychological similarities, and these were classified separately.

### Results

#### Participants

Participants’ characteristics were as follows: mean number of years of experience (*M*_exp_) = 12.82 ± 10.20 years; mean number of correct responses to knowledge questions (*M*_corr_) = 14.29 ± 1.57. The participants were divided into two groups based on the following two criteria: whether they possessed the credentials required to diagnose patients, and whether they were involved with children in their professional work. Ten participants were medical doctors with the credentials required to diagnose patients (e.g., pediatricians, pediatric neurologists, and psychiatrists: 5 women, 5 men; *M*_age_ = 52.30 ± 12.72 years; *M*_exp_ = 13.48 ± 5.75 years; *M*_corr_ = 13.80 ± 2.15), and 24 were other professionals who provided developmental support and did not possess the credentials necessary to diagnose patients (e.g., psychotherapists, speech therapists, and social workers: 12 women, 12 men; *M*_age_ = 39.17 ± 12.68 years, *M*_exp_ = 12.54 ± 11.67 years, *M*_corr_ = 14.50 ± 1.25).

The main effect of group was significant with respect to age, *t*(32) = 2.8, *p* = 0.010, *d* = 1.04, as medical doctors’ mean age was higher than that of other professionals; however, there were no significant differences in years of experience, *t*(31) = 0.32, *p* = 0.80, *d* = 0.09, or numbers of correct responses to knowledge questions, *t*(12) = 0.96, *p* = 0.40, *d* = 0.45, between groups. Because the study included so few participants, and the accuracy of participants’ knowledge did not differ significantly between the two groups, we analyzed the responses from the two groups together.

#### Conceptual Understanding

Three participants were excluded from the analysis because their reasons for their choices were unclear. The frequencies for the four statements differed significantly and we found a large effect size, χ^2^(3) = 23.58, *p* < 0.001, *w* = 0.872, power = 0.990; *different* = 61%, *differentiation unnecessary* = 23%, *same* = 10%, and *not disorders* = 6%. The *different* statement was chosen more frequently than *same* and *not disorders* (*same*: *p* = 0.003, *not disorders*: *p* = 0.001; *p*-values were adjusted using Benjamini-Hochberg procedures); however, the difference between *different* and *differentiation unnecessary* only approached significance (*p* = 0.058). The frequencies for the other three statements did not differ significantly, *p*s > 0.10. In addition, the numbers of participants who did and did not select the *different* statement (i.e., the total number for the other three statements) did not differ significantly and the effect size was small [χ^2^(1) = 1.581, *p* = 0.209, *w* = 0.226, power = 0.242]. The results regarding the free descriptions are shown in **Table 1**.

#### Psychological Similarities and Differences

Responses that had unclear meanings or did not conform to the questions were excluded. Therefore, data from four participants were excluded from the analysis of psychological similarities and three participants were excluded from the analysis of psychological differences.

**Table 1:** Reasons for choices regarding conceptual understanding (*N* = 34).

Response category	*f*	%
**Different**	19	61
Appearances or characteristics differ	7	23
Interventions differ	7	23
Brain activity differs	3	10
Other (by elimination)	2	6
**Differentiation unnecessary**	7	23
Individual support is required despite diagnosis	4	13
The high comorbidity rate	1	3
Lack of evidence that distinguishes between the disorders	1	3
They have something in common	1	3
**Same**	3	10
Both ADHD and ASD involve developmental imbalance	2	6
ADHD and ASD are on the same spectrum	1	3
**Not disorder**	2	6
ADHD and ASD are characteristics that will not improve	1	3
ADHD and ASD will improve	1	3

*ADHD, attention deficit hyperactivity disorder; ASD, autism spectrum disorder. No multiple responses; three missing responses*.

#### Similarities

The analysis of psychological similarities included data for 30 participants. The summaries regarding psychological similarities concerned “overt characteristics” (87.3%), “secondary issues” (7.3%), and “intervention” (5.5%; **Table 2** and Supplementary Table S1). The “overt characteristics” summary included similarities in observed clinical conditions, such as cognitive problems and communication difficulties; the “secondary issues” summary included deuteropathies, which were mainly internal problems other than cardinal symptoms; and the “intervention” summary included effective interventions for both disorders. Most responses concerned “overt characteristics.” In particular, “difficulty understanding non-verbal information and/or situations” was chosen more frequently than the other responses (16.4%).

**Table 2:** Psychological similarities between ADHD and ASD (*N* = 34).

Response category	*f*	%
**Overt characteristics**		
Difficulty understanding non-verbal information and/or situations	9	16.4
Issues involving emotion	6	10.9
Issues involving cooperation with others	5	9.1
Difficulty communicating	4	7.3
Deviation of interest or concern	4	7.3
Issues involving executive function	3	5.5
Poor flexibility	3	5.5
Self-control difficulties	3	5.5
Other overt characteristics	11	20.0
**Secondary issues**		
Decline in self-evaluation	4	7.3
**Intervention**		
Effectiveness of environmental regulation	3	5.5
**Total**	55	100.0

*ADHD, attention deficit hyperactivity disorder; ASD, autism spectrum disorder. Multiple responses were valid; four missing responses*.

#### Differences

The analysis of psychological differences included data for 31 participants. The summaries regarding psychological differences concerned “overt characteristics” (63.5%), “causes” (14.6%), “interventions” (7.3%), “secondary issues” (4.9%), and “other” (9.7%; **Table 3**). The “overt characteristics,” “secondary issues,” and “intervention” summaries included the responses observed for similarities, and “causes” concerned the causes of overt characteristics. Four responses clearly indicated that ADHD involved more serious issues, relative to those involved in ASD (represented by the number of responses indicating that the issues described in the items were most serious in ASD), and 14 suggested that ASD involved more serious issues than those involved in ADHD (represented by the number of responses indicating that the issues described in the items were most serious in ADHD; **Table 3**). A significantly higher number of responses suggested that serious difficulties were more numerous in ASD (i.e., 14) than in ADHD and the effect size was large [i.e., 4; χ^2^(1) = 5.556, *p* = 0.018, *w* = 0.556, power = 0.872].

**Table 3:** Psychological differences between ADHD and ASD (*N* = 34).

Response category	*f*	%	More serious in		Example
			ADHD^a^	ASD^b^	
**Overt characteristics**					
Interaction with others	6	14.6	0	5	It is easier to build empathic relationships in ADHD than it is in ASD [other professional]
Emotion	3	7.3	0	0	Individuals with ADHD rapidly express their emotion and/or behavior [other professional]
Attention	2	4.9	1	0	Individuals with ADHD are easily distracted [other professional]
Characteristic leading to trouble	2	4.9	0	0	ADHD is a behavioral issue, and ASD involves multiple issues such as those involving behavior, cognition, and emotion [medical doctor]
Persistence	2	4.9	0	2	ASD is more persistent than ADHD, and there is greater difficulty in adapting to change [other professional]
Reaction to difficult situations	2	4.9	1	1	When individuals feel anxious, the switch from anxiety occurs quickly in ADHD and slowly in ASD [other professional]; Individuals with ASD might perform daily life tasks more effectively than those with ADHD can because, when they do not understand the content of a conversation, it does not affect them as much as someone with ADHD, who can easily become panicked in these situations [medical doctor]
Self-understanding	2	4.9	0	0	Individuals with ADHD have an omnipotent feeling toward themselves; however, those with ASD do not [other professional]
Other	7	17.1	1	4	Individuals with ADHD are more hyperactive relative to those with ASD [other professional]; Individuals with ADHD seem to be rather open to understand the situation [medical doctor]
**Causes**					
Factors causing issues in interpersonal relationships	5	12.2	0	0	Difficulties in understanding conversations or situations are caused by inattention or impulsivity in ADHD and persistence or lack of imagination in ASD [medical doctor]
Factors causing issues involving executive function or the central nervous system	1	2.4	0	0	Issues involving executive function or the central nervous system are caused by impulsivity in ADHD and lack of imagination in ASD [other professional]
**Interventions**					
Screening	2	4.9	0	0	ASD becomes obvious during early childhood [medical doctor]
Treatment	1	2.4	0	1	People with ADHD can control their behavior to some extent by taking medicine [other professional]
**Secondary issues**					
Self-evaluation	2	4.9	1	1	It is easier to decrease self-evaluation in ASD [medical doctor]; ADHD presents low self-evaluation earlier relative to ASD [other professional]
**Other**					
There is no difference	2	4.9	0	0	ADHD and ASD do not differ much [other professional]; There are no differences between ASD and ADHD [medical doctor]
Completely different	1	2.4	0	0	ADHD and ASD are completely different disorders [other professional]
I do not know	1	2.4	0	0	It is impossible to determine whether differences in characteristics depend on differences between individuals or the disorders [other professional]
**Total**	41	100.0	4	14	

*ADHD, attention deficit hyperactivity disorder; ASD, autism spectrum disorder. Multiple responses were valid; three missing responses; ^*a*^“More serious in ADHD” = the number of descriptive responses indicated that the issues were more serious in ADHD relative to ASD; ^*b*^“More serious in ASD” = the number of descriptive responses indicated that the issues were more serious in ASD relative to ADHD*.

#### Biological Similarities and Differences

As with conceptual understanding, data for three participants were excluded from the analysis of biological similarities and differences. There were no multiple responses.

#### Similarities

Responses regarding biological similarities were classified into five categories: “brain issues,” which concerned issues regarding brain activity, neurotransmitters, and functional and/or structural problems in the brain (32.3% for all); “brain issues and genetic factors,” which concerned ADHD- and ASD-related problems regarding brain activity and genetic factors (12.9%); “genetic factors,” which concerned the ease that both ADHD and ASD are inherited (9.7%); “other,” which included hypersensitivity (9.7%); and “nothing,” which indicated that participants saw no similarities or did not know (35.5%).

#### Differences

Responses concerning biological differences were classified into three categories: “brain region with problem,” “other,” and “nothing.” The “brain region with problem” category included problems involving the prefrontal region in ADHD and multiple regions in ASD; however, 12 out of 14 responses did not indicate detailed regional differences (45.2% for all). The “other” category included the belief that ADHD symptoms would reduce with development, but ASD symptoms would not, or the belief that ADHD was likely to involve comorbidity, but ASD was not (9.7%). “Nothing” indicated that participants saw no differences or did not know (45.2%).

#### Biological Factors and Conceptual Understanding

Participants were divided into two groups unlike psychological similarities and differences: those who believed in certain biological factors and those who did not. The number of participants who saw similarities and differences between the two disorders in the group that believed in certain biological factors did not differ significantly relative to that observed in the group that did not believe in certain biological factors and the effect size was small, especially regarding biological difference [similarities: χ^2^(1) = 2.613, *p* = 0.106, *w* = 0.290, power = 0.365; differences: χ^2^(1) = 0.290, *p* = 0 .590, *w* = 0.097, power = 0.084). We performed chi-square tests to analyze the relationships between the frequency with which *different* was chosen for conceptual understanding and recognition of the presence of biological similarities and differences (**Table 4**). Recognition of the relationship between ADHD and ASD was not significantly associated with recognition of biological similarities, χ^2^(1) = 0.056, *p* = 0.813, *w* = 0.119, power = 0.098, nor with biological differences, χ^2^(1) = 0.276, *p* = 0.599, *w* = 0.170, power = 0.150. The frequency of participants who believed in certain biological differences did not differ between participants who did and did not choose the *different* statement.

**Table 4:** The Relationship between biological factors and conceptual understanding (*N* = 29).

	Similarities		Differences	
	Exist	Do not exist	Exist	Do not exist
**Selected *different***
Observed	11	8	11	8
Expected	11.8	7.2	9.8	9.2
**Did not select *different***
Observed	7	3	4	6
Expected	6.2	3.8	5.2	4.8

*The “Exist” column shows responses from participants who believed in certain biological factors and the “Do not exist” column shows responses from participants who did not believe in certain biological factors*.

#### Diagnoses

The mean diagnosis rates were as follows: ADHD = 28.41 ± 13.45, ASD = 49.00 ± 13.66, and ADHD + ASD = 23.52 ± 12.97. The results of the one-way ANOVA showed that the main effect of diagnosis was significant, *F*(2,64) = 22.78, *p* < 0.001, η_p_^2^ = 0.416. The diagnosis rate for ASD was significantly higher than that for ADHD (ADHD: *p* < 0.001, *d* = 1.52) and ADHD + ASD (*p* < 0.001, *d* = 1.91). There was no significant difference between diagnosis rates for ADHD and ADHD + ASD (*p* = 0.569, *d* = 0.37).

### Discussion

The aim of the study was to determine whether and how bias in the understanding of ADHD and ASD existed in the Japanese population. The results showed that participants focused more closely on difficulties concerning ASD, relative to those concerning ADHD, in their free descriptions regarding psychological differences. Further, the diagnosis rate for ASD was significantly higher than those for ADHD and ADHD + ASD. This finding differs from previous studies, in which the prevalence rate for ADHD was higher than that of ASD (Matson and Kozlowski, 2011; Willcutt, 2012; American Psychiatric Association [APA], 2013; Thomas et al., 2015). However, the results were consistent with those reported by The National Institute of Special Needs Education Japan [NISE] (2015). In addition, the numbers of participants who did and did not consider ADHD and ASD as distinct disorders (i.e., those who did and did not choose the *different* statement) did not differ significantly. Further, the frequency of participants who believed in biological similarities and differences did not differ markedly from the participants who did not believe in biological factors, unlike psychological factors. The results are discussed in detail below.

The results showed that participants focused more strongly on the overt characteristics of ASD (i.e., difficulty understanding non-verbal information or situations, problems cooperating with others, poor flexibility, difficulty in communication, and deviation of interests or concerns) relative to symptoms such as ADHD-related inattention, impulsivity, and hyperactivity, when considering the psychological similarities of ADHD and ASD. Furthermore, participants referred to ASD more frequently than ADHD in the free descriptions of the psychological differences between ADHD and ASD. These results are consistent with our hypothesis that participants would focus on non-verbal communication skills. Based on previous studies that showed individuals with ASD presented with ADHD-related impulsivity and inattention (Nicolson and Castellanos, 2000) and those with ADHD presented with ASD-related symptoms (Nijmeijer et al., 2009), it was natural to expect that participants’ focus on ADHD-related characteristics would be as strong as that on ASD-related characteristics. However, the results suggested that, relative to ADHD characteristics, participants were more sensitive to ASD characteristics and focused on them most strongly.

The results also showed that participants’ recognition of the relationship between ADHD and ASD did not necessarily correspond to operational diagnostic criteria. Even though ADHD and ASD are similar in that they are both neurodevelopmental disorders, they are classified as separate disorders with distinct diagnostic criteria. Participants who chose the *different* statement believed that ADHD- and ASD-related characteristics and/or interventions differed. However, some participants focused on the need for individualized support or believed that the disorders were on the same spectrum. It is possible that recognition of individualized treatment needs prevented participants from distinguishing between ADHD and ASD, and this, in addition to the above-mentioned biases, led to weak diagnostic agreement. Considering the insufficient knowledge about biological factors, greater clarification and dissemination of information regarding the biological similarities, differences, and overlap between the disorders could be useful in improving differential diagnoses for ADHD and ASD and enhance evaluation tools designed to determine whether individuals have one or both disorders.

If Japanese people are biased, in that they find it easy to focus on ASD-related characteristics specifically and separate them from the multiple other possible symptoms, this could affect the accuracy of assessments and lead to weak diagnostic agreement (i.e., over-diagnosis of ASD and under-diagnosis of symptoms). Therefore, we conducted a second experiment to determine whether participants exhibited sensitivity to ASD.

## Study 2

### Bias in Assessing Children’s Behavior via a Fictional Assessment Task

Based on the results of Study 1, we assumed that the participants, who could affect the assessment of children’s development, were more sensitive to ASD symptoms than those of ADHD. However, the results of Study 1 did not explain how this bias affected assessment. If participants were sensitive to ASD-related symptoms in examining children’s behavioral or emotional characteristics, they could have regarded ADHD-related characteristics as signs of ASD. Moreover, they could have suspected that mental and behavioral symptoms that were similar to those observed in ADHD in the diagnostic criteria were related to ASD rather than ADHD.

For example, the DSM-IV-TR (American Psychiatric Association [APA], 2000) and DSM-5 (American Psychiatric Association [APA], 2013) both indicate that hyperactivity, impulsivity, and inattention in ADHD are difficult to differentiate from the behavior of typically active children, children with oppositional defiant disorder (ODD), and children with disinhibited social engagement disorder (DSED). ODD is a classified as one of the disruptive, impulse-control, and conduct disorders and characterized by a pattern of angry/irritable mood, argumentative/defiant behavior, and vindictiveness, and differential diagnosis between ODD and other disorders, including ADHD, is required. DSED is one of the trauma- and stressor-related disorders and characterized by a pattern of behavior whereby children actively approach and interact with unfamiliar adults, and differential diagnosis between DSED and ADHD is required.

These characteristics tend to be difficult to distinguish from those of ADHD. However, if there is bias in the tendency to attribute symptoms to ASD, these characteristics could be more likely to be confused with ASD than ADHD. In Study 2, we examined clinicians’ assessment tendencies using the original assessment task.

We assumed that participants in Study 1 recognized the similarities and difficulties involved in differentiating ADHD from other mental problems; however, it was possible that participants were unaware of them, even though bias was observed for ADHD and ASD. Therefore, we confirmed subjective similarities and related difficulties in differentiation.

### Method

#### Participants

We searched for three or four specialized agencies in the mental support field for children (e.g., hospitals and the Society of Certified Clinical Psychologists) per prefecture. All Societies of Certified Clinical Psychologists in Japan were included, because each prefecture contained a society. In the hospital selection process, we chose larger hospitals (e.g., university and general hospitals) because we assumed that they employed more medical doctors than private hospitals. In prefectures with numerous hospitals, we selected large hospitals randomly.

Written requests for responses to a web survey, which was conducted via Qualtrics.com, were sent to 164 agencies. The agencies and their constituents received information regarding the study purpose, the voluntary nature of participation, and anonymity, as in Study 1. The study was conducted between October 2014 and April 2015.

Fifty-three medical doctors who specialized in children’s mental health and clinical psychologists who supported children at elementary school, and who had not participated in Study 1, participated in Study 2 (35 women, 18 men; *M*_age_ = 39.23 ± 10.34 years, *M*_exp_ = 9.10 ± 6.94 years). Participants consisted of 12 pediatricians and psychiatrists (6 women, 6 men; *M*_age_ = 42.83 ± 9.06 years, *M*_exp_ = 10.50 ± 7.35 years) and 41 clinical psychologists (29 women, 12 men; *M*_age_ = 38.17 ± 10.56 years, *M*_exp_ = 8.69 ± 6.85 years).

#### Questionnaire Content

We examined assessment task results, similarity and difficulty ratings, and the diagnostic criteria usually used by participants. We used terms based on the DSM-IV-TR, rather than the DSM-5, for the reasons described in Study 1. Therefore, ASD was referred to as pervasive developmental disorder, and DSED was referred to as a reactive attachment disorder (RAD) in the web-based research.

#### Assessment Task

Participants were asked to read four fictional cases (see Supplement 1 of Supplementary Materials) and diagnose the mental health problems that manifested as internal and/or external problems for each case. The instructions were as follows: “The following four questions are about fifth-year pupils’ behavior at school; the pupils are enrolled in a general education class at elementary school. If you suspect mental health or behavioral problems for each child, what do you suspect? If you feel that there is insufficient information available for assessment, please imagine what you suspect as the potential problem, using the information provided, and engage in subsequent information gathering and support provision. There are various possible responses to the four questions, and duplicate responses are permitted.” We used the phrase “mental health or behavioral problems” rather than “mental health problems” alone to provide participants with images of internal and/or external problems from the vignettes. Assumptions of comorbidity and multiple responses were permitted, and participants were asked to prioritize multiple responses.

#### Similarity and Difficulty Ratings

Participants rated the similarities and difficulties involved in distinguishing between ADHD and other mental health problems. In the similarity ratings, participants were asked to indicate the extent to which they felt ADHD was similar to certain other conditions (i.e., ASD, ODD, and DSED), using a seven-point Likert scale ranging from 1 (*extremely dissimilar*) to 7 (*extremely similar*). In the difficulty ratings, participants were asked to indicate the extent of the difficulty they experienced in distinguishing between ADHD and other problems (i.e., ASD, ODD, DSED, hyperactivity, impulsivity, and inattention), using a seven-point Likert scale ranging from 1 (*very easy*) to 7 (*very difficult*). In both tasks, participants chose “unknown” if they had not previously heard of a disorder.

To prevent the similarity and difficulty rating tasks from influencing the assessment task, we included five dummy comparison disorders (i.e., anxiety disorder, bipolar disorder, conduct disorder, intellectual disabilities, and depressive disorders). Prior to the rating exercise, we asked participants to indicate whether they had ever heard of the eight above-mentioned disorders by selecting either “I have heard of it” or “I haven’t heard of it.”

#### Diagnostic Criteria

To confirm whether assessment standards were consistent among participants, we examined the diagnostic criteria (definitions) participants usually used to assess mental health or behavioral problems in children. Participants were asked to number the following criteria in ascending order according to usage: DSM-IV-TR, DSM-5, International Statistical Classification of Diseases, Tenth Revision (ICD-10), and other (describe explicitly).

#### Compliance With Ethical Standards

All study participants provided informed consent. Ethical approval was not required for the study.

#### Data Analyses

Statistical analyses were performed using R3.4.0 for Mac OS (R Core Team, 2017) and statistical power was calculated using G^∗^Power 3.1 (Faul et al., 2009). In the analysis of the assessment task, responses of “autism,” “ASD,” “pervasive developmental disorder,” and “Asperger’s syndrome” were considered correct responses to the question regarding the case involving ASD. “Disinhibited RAD,” which is the term used to describe DSED in the DSM-IV-TR, “RAD,” which is the superordinate category for disinhibited RAD, and “attachment disorder,” which is the superordinate concept for RAD, were considered correct responses to the question regarding the case involving DSED. Descriptions of comorbidity and secondary issues that included correct responses were also considered correct. Accuracy in the four tasks was compared using Cochran’s *Q* test. Binomial tests were performed to compare the numbers of correct and incorrect responses.

We excluded six participants who chose “I haven’t heard of it” for at least one disorder in the similarity and difficulty ratings. One participant had not heard of DSED or ODD, four had not heard of DSED, one had not heard of ODD, and one had not heard of conduct disorder. The Friedman test and multiple comparisons of the Wilcoxon signed-rank test (Bonferroni method) were used to compare rating values among eight similarity characteristics and 11 difficulty characteristics.

### Results

#### Assessment of Fictional Cases Task

The correct response rates were 86.8, 92.5, 32.1, and 34.0% for the questions regarding cases involving ADHD, ASD, DSED, and ODD, respectively (**Table 5**). Accuracy in the four tasks differed significantly, Cochran’s *Q*(3) = 71.45, *p* < 0.001. The results of McNemar’s chi-squared test showed that the accuracy of responses to questions regarding cases involving ASD and ADHD was higher than that for questions regarding cases involving ODD and DSED, ASD vs. ODD: χ^2^(1) = 29.03; ASD vs. DSED: χ^2^(1) = 26.69; ADHD vs. ODD: χ^2^(1) = 26.04, ADHD vs. DSED: χ^2^(1) = 25.29, all *p*s < 0.001. The accuracy of responses did not differ significantly between cases involving ASD and ADHD, χ^2^(1) = 0.44, *p* = 0.505, or ODD and DSED, χ^2^(1) < 0.001, *p* > 0.999.

**Table 5:** Responses, frequencies, and percentages in the assessment task (*N* = 53).

Response category	*f*	%
**ADHD**		


ADHD	46	86.8


Intellectual disability	2	3.8


ASD	1	1.9


Developmental issues	1	1.9


Impulsivity and inattentiveness	1	1.9


Learning disorder	1	1.9


Psychological stress	1	1.9


**ASD**		


ASD	49	92.5


Developmental issues	2	3.8


Attachment disorder	1	1.9


Developmental disorder	1	1.9


**DSED**		


ASD	20	37.7


DSED	17	32.1


ADHD	3	5.7


Intellectual disability	3	5.7


Developmental disorder	2	3.8


Developmental issues	2	3.8


Issues concerning social distance	2	3.8


Typical development	2	3.8


Comorbid ADHD and ASD	1	1.9


Poor social skills	1	1.9


**ODD**		


ODD	18	34.0


Attachment disorder	9	17.0


Child abuse	5	9.4


Domestic issue	5	9.4


ADHD	3	5.7


ASD	3	5.7


Issues in relationships with adults	3	5.7


Antisocial behavior	1	1.9


Comorbid ADHD and ASD	1	1.9


Conduct disorder	1	1.9


Developmental disorder	1	1.9


Neurosis	1	1.9


Issues concerning lack of nurturing	1	1.9


Psychological stress	1	1.9

*ADHD, attention deficit hyperactivity disorder; ASD, autism spectrum disorder; DSED, disinhibited social engagement disorder; ODD, oppositional defiant disorder. The ASD response category included the following responses: autism, autism spectrum disorder, pervasive developmental disorder, and Asperger’s syndrome. The DSED response category included disinhibited reactive attachment disorder, attachment disorder, and reactive attachment disorder*.

The numbers of correct responses to questions regarding cases involving ADHD (binomial test, *p* < 0.001) and ASD (*p* < 0.001) were significantly higher than numbers of incorrect responses. In addition, the number of incorrect responses to questions regarding cases involving ODD (*p* = 0.027) and DSED (*p* = 0.013) was significantly higher than the number of correct responses. Furthermore, the most common incorrect response to the question regarding the case involving DSED was “ASD” (37.7%).

### Similarity and Difficulty

Means and *SD*s for similarity and difficulty are presented in Supplementary Table S2. The Friedman test showed that the ratings for similarity and difficulty differed significantly between the target and dummy disorders, similarity: χ^2^(7) = 136.70, *p* < 0.001, η^2^ = 0.416, power > 0.999; difficulty: χ^2^(10) = 98.83, *p* < 0.001, η^2^ = 0.210, power = 0.999. Multiple comparisons of the Wilcoxon signed-rank test (Bonferroni method) indicated that the mean values for ASD, ODD, and DSED were significantly higher than those for anxiety disorder (*p*s < 0.05), depressive disorders (*p*s < 0.01), and intellectual disabilities (*p*s < 0.05), for both similarity and difficulty (see Supplementary Table S3). There were no significant differences among mean values for ASD, ODD, and DSED.

### Criterion

The numbers of participants who used the DSM-IV-TR, DSM-5, ICD-10, and other criteria were 24 (45.28%), 20 (37.74%), five (9.43%), and four (7.55%; e.g., “multiple decision”), respectively.

### Discussion

To determine whether medical doctors and school counselors who supported school-age children were more sensitive to ASD-related than ADHD-related characteristics, we administered a questionnaire involving four assessment tasks (the correct responses were “ASD,” “ODD,” “DSED,” and “ADHD”) and examined subjective rates of similarity and difficulty in distinguishing between ADHD and other mental health problems. In the assessment task, accuracy rates for cases involving ASD and ADHD were higher than those for cases involving ODD and DSED. Furthermore, the case involving DSED was assessed mainly as ASD, rather than DSED or ADHD. The results also showed that participants considered ASD, ODD, and DSED as similar to ADHD. Moreover, although the criteria used by participants daily could have influenced assessment results, almost all participants based their assessments on common operational diagnostic criteria.

The numbers of correct responses to questions regarding cases involving ASD and ADHD were higher than the numbers of incorrect responses. Based on the results of Study 1, we hypothesized that ADHD would be confused with ASD; however, this hypothesis was not supported. This finding could have occurred because we used typical behavioral characteristics in the fictional cases, and participants were likely to diagnose ADHD, rather than ASD, when children presented with typical ADHD-related characteristics.

The similarity and difficulty ratings indicated that DSED was considered similar to ADHD, and participants found it difficult to distinguish between these two disorders. This accords with the DSM-5 (American Psychiatric Association [APA], 2013), which states that differential diagnoses are required between DSED and ADHD and between ASD and RAD, which is a trauma- and stressor-related disorder. Based on the DSM-5 and their understanding regarding the similarity of DSED and ADHD, participants were expected to confuse DSED with ADHD in the assessment task; however, they confused DSED with ASD rather than ADHD. This result indicates that, notwithstanding participants’ understanding of the similarity between DSED and ADHD, they mistakenly assessed the related behavioral characteristics as ASD. Therefore, there was a dissociation between participants’ knowledge and behavior, which affected assessment accuracy.

Although clinicians showed elevated levels of accuracy in differentiating between ADHD and ASD, our original concern remained. In practice, children present with various characteristics including normal activity, inattention, depression, and strong preferences; from these, mental health professionals are usually required to detect abnormal traits. However, considering participants’ difficulties in diagnosing DSED, it is possible that they were biased toward ASD-related features in their assessment of children with not only hyperactivity, impulsivity, and inattention but also interpersonal problems.

In addition, considering the high accuracy rates for ADHD and ASD assessments and low accuracy rates for ODD and DSED assessments, differences in familiarity with developmental and other psychiatric disorders could have influenced the assessment results. One probable reason for this finding is that participants could have been more familiar with and likely to diagnose developmental disorder relative to other disorders, as a few participants had not heard of “DSED,” “ODD,” or “conduct disorder.” When unsure, the participants might assess children’s behavior as ASD because they are more familiar with it.

## General Discussion

The results of the study showed inconsistencies and biases in understanding regarding ADHD and ASD in individuals who performed psychological assessments in the Japanese population. Almost half of the participants in Study 1 understood that ADHD and ASD are distinct disorders; however, the remaining participants did not. In addition, participants in Study 1 focused more strongly on ASD relative to ADHD. Moreover, based on the results of Study 2, mental health professionals could exhibit a tendency toward confusing DSED with ASD.

The service climate, with respect to medicine, education, and welfare, in each country should be considered when discussing biases in assessment and diagnosis. To diagnose children with ASD is not considered to provide greater benefits than diagnosing other mental health disorders, including ADHD, in Japan, as children with any type of mental health disorder are almost equally eligible to receive welfare, medical, and educational services; mental disability certificates for the provision of financial aid; pharmacotherapy; and social skills training. However, despite this equality in the service climate, we conclude that the attitudes of medical doctors and other professionals could be biased toward ASD, as the participants’ focus on ASD-related characteristics was stronger than that on ADHD- and DSED-related characteristics in both experiments.

The rating of child hyperactive-disruptive behaviors by mental health professionals differs per culture (Mann et al., 1992); therefore, distinct cultures have diverse social norms regarding valuable behavior. For example, harmony and cohesiveness in the group is valuable in non-Western cultures; in contrast, Western cultures emphasize autonomy (Chen and French, 2008). These distinct values may affect children’s education and development (Chen and French, 2008), which would also influence assessment tendencies in clinicians.

Matson et al. (2011, 2017) examined children’s autism as rated by parents, guardians, caretakers, or teachers in Israel, South Korea, the United Kingdom, and the United States (Matson et al., 2011) and in Greece, Italy, Japan, Poland, and the United States (Matson et al., 2017). Their multinational comparisons consistently showed that ratings made by participants differed among multinational groups. They found that item endorsement frequencies for the “Verbal Communication” subscale were consistently the highest for children from the United States and Poland, whereas there was greater variation for the “Repetitive Behaviors/Restricted Interest” subscale (Matson et al., 2017). On the “Socialization/Non-verbal Communication” subscale, there was some variation across items. Interestingly, the children from Japan had the highest rate compared with other countries for the items, “Understanding age appropriate jokes, figures of speech, or sayings,” “Use of too few or too many social gestures,” and “Intellectual abilities,” even though children from the United States and Poland generally had higher endorsement frequency for these items. This is consistent with our result that participants focused on children’s social interactions. The current results indicate that the behavior aspect of individuals with ASD, which are sometimes considered as deficits, for example with regards to interaction with others, are seen as more valuable than other psychological characteristics in group-oriented social contexts.

Some studies of psychological assessment have noted the impact of cultural differences via internationalization. Main concerns included how children’s ethnic diversity affects mental health professionals and/or teachers’ decisions regarding participants’ behavior (e.g., Reid et al., 1998), how mental health professionals and/or teachers’ cultural differences affect their decisions regarding participants’ behavior (e.g., Mann et al., 1992; Matson et al., 2011), and the interaction between raters and children’s ethnicity (de Ramírez and Shapiro, 2005). For example, Reid et al. (1998) found that teachers’ ratings of ADHD for African-American children were significantly higher than those for white children. They noted that teachers’ negative cognitive bias for a specific ethnicity could be caused by the halo effect, which led to high ratings of ADHD. Mann et al. (1992) found that perceptions of hyperactivity vary significantly across raters’ countries, even if uniform rating criteria are applied. The current study indicates that future research should also focus on the clinician’s cultural background to determine if it affects clinical decision-making regarding disease identification.

Regarding bias in assessment, Bruchmüller et al. (2012) found that therapists exhibited a bias toward diagnosing ADHD in boys rather than girls. According to the current results and those of Bruchmüller et al. (2012), mental health professionals require training to reduce the influence of their own biases. However, this could be problematic, as therapists could find it difficult to avoid the influence of multiple factors, such as their own personalities or cultural contexts, leading to errors in assessment. As mentioned in the Introduction, heuristics, which constitute one of the factors affecting clinical judgment, play a key role in the reasoning process (Vázquez-Costa and Costa-Alcaraz, 2013). Identifying and understanding the characteristics of heuristics and implementing strategies, such as metacognitive training and recognition of difficulties in diagnosis, are critical to avoid this type of cognitive error (Vázquez-Costa and Costa-Alcaraz, 2013). In addition, the results indicate that bias based on cultural background in doctors and therapists could affect their clinical judgment.

When therapists assess children’s psychiatric traits, they usually use multiple assessment methods and test batteries (e.g., observation, questionnaires, clinical interviews with caregivers or teachers, psychological and neuropsychological tests, and electroencephalography). When assessing ASD or ADHD, diagnostic schedules and questionnaires (e.g., the Autism Diagnostic Interview-Revised [Le Couteur et al., 1989], Diagnostic Interview for Social and Communication Disorders, [Wing et al., 2002], Autism Spectrum Screening Questionnaire [Ehlers et al., 1999], and ADHD Rating Scale-IV [DuPaul et al., 1998]) are vital tools that are used to collect and share information, particularly that regarding children. However, these assessment tools do not consider therapist bias. The development of assessment strategies is urgently required to support professionals in overcoming their own biases. In addition to ensuring that therapists listen to clients’ descriptions of their symptoms and measure these symptoms thoroughly, efforts to address therapists’ tendency to focus on the detection of specific characteristics (e.g., rating therapists’ tendency to focus on ASD-related traits, as clients’ ASD scores could be affected by therapists’ biases) could improve assessment accuracy.

The current study was subject to some limitations. For example, the sample size was small, perhaps because few professionals and professional medical organizations are able to diagnose or assess developmental disorders (The Administrative Evaluation Bureau, 2017), and we limited participants’ occupational categories based on our interests (i.e., mental health professionals). Therefore, there were very few qualified individuals in the specialized agencies invited to participate in the study. In addition, the small sample size prevented the comparison of occupational categories. Further, we used only one vignette per diagnosis in Study 2; therefore, future studies should use experimental designs that include multiple trials (i.e., vignettes) for each diagnosis. Moreover, the survey included only Japanese professionals. Therefore, the results are not generalizable to other non-Western cultural contexts or directly comparable to Western cultures.

Despite these limitations, the results indicated that doctors’ and psychological therapists’ cultural backgrounds affected their assessments. It is necessary to examine these issues in other countries, because cultural or regional differences in prevalence and increases in the incidence of mental health problems have been observed (Matson and Kozlowski, 2011; Getahun et al., 2013) and could depend on various biases, and it is possible that other mental health issues reflect distinct cultural contexts. Furthermore, as this cultural effect on clinical judgment might occur for other psychological disorders in addition to ADHD and ASD, it is important that cultural effect of relevance to each disorder are investigated. Finally, as Japanese individuals frequently communicate elliptically, while Western individuals express themselves clearly (Ray, 1998), to understand the former requires the ability to “take a hint” or “read between the lines” during communication. Understanding Japanese individual’s intentions is relatively difficult, as subjective well-being is closely associated with interdependence and interpersonal engagement in Japan, but with the frequency of interpersonally disengaged positive emotions (e.g., pride) in the United States (Kitayama et al., 2000). Moreover, because Asian people characteristically attach high value to non-verbal information (Hall, 1976; Ishii et al., 2003), it is possible that a given individual’s unusual behavior would be attributed to non-verbal issues. The current results suggest that the knowledge of cultural differences in communication style or social norms could contribute to the understanding of clinical diagnoses.

## Ethics Statement

After careful consideration, we have concluded that our survey study does not require an ethical review. There are two main reasons for this conclusion. First, the content of the study has been approved by the respective developmental support field agencies. Participants were invited from specialized agencies in the developmental support field (hospitals, elementary schools, child consultation centers, and Support Center for Developmental Disorders). Each agency was informed about the purpose of the research, voluntary nature of participation, and anonymity of the responses collected. The agencies could inform its constituents. Therefore, participants were recruited only if the agency confirmed that the study was appropriate for its constituents. Second, the same details were provided to the participants. Before participants answered the questionnaire, we set opportunities at different times for agencies or participants to choose whether they wanted to join the study. In addition, participants were allowed to quit answering the questionnaire anytime they wanted. Although our study has not been approved by the research ethics committee, we believe that the voluntary participation and effort of participants is significant. Therefore, our study complies with the ethical policy of your journal.

## Author Contributions

MM, SK, and MN: Study conception or design, interpretation of study data, drafting the manuscript or revising it for critical intellectual content, final approval of the version to be published, and agreement to be accountable for all aspects of the work in ensuring that questions related to the accuracy or integrity of any part of the work are appropriately investigated and resolved. MM: acquisition and analysis.

## Conflict of Interest Statement

The authors declare that the research was conducted in the absence of any commercial or financial relationships that could be construed as a potential conflict of interest.

## References

[B1] American Psychiatric Association [APA] (2000). *Diagnostic and Statistical Manual of Mental Disorders: DSM-IV-TR.* Washington, DC: American Psychiatric Publishing.

[B2] American Psychiatric Association [APA] (2013). *Diagnostic and Statistical Manual of Mental Disorders: DSM-5.* Washington, DC: American Psychiatric Publishing.

[B3] Brieber S. Neufang S. Bruning N. Kamp-Becker I. Remschmidt H. Herpertz-Dahlmann B. (2007). Structural brain abnormalities in adolescents with autism spectrum disorder and patients with attention deficit/hyperactivity disorder. *J. Child Psychol. Psychiatry* 48 1251–1258. 10.1111/j.1469-7610.2007.01799.x 18093031

[B4] Bruchmüller K. Margraf J. Schneider S. (2012). Is ADHD diagnosed in accord with diagnostic criteria? Overdiagnosis and influence of client gender on diagnosis. *J. Consult. Clin. Psychol.* 80 128–138. 10.1037/a0026582 22201328

[B5] Chen X. French D. C. (2008). Children’s social competence in cultural context. *Annu. Rev. Psychol.* 59 591–616. 10.1146/annurev.psych.59.103006.093606 18154504

[B6] Conrad P. Bergey M. R. (2014). The impending globalization of ADHD: notes on the expansion and growth of a medicalized disorder. *Soc. Sci. Med.* 122 31–43. 10.1016/j.socscimed.2014.10.019 25441315

[B7] Daley T. C. (2002). The need for cross-cultural research on the pervasive developmental disorders. *Transcult. Psychiatry* 39 531–550. 10.1177/136346150203900409

[B8] de Ramírez R. D. Shapiro E. S. (2005). Effects of student ethnicity on judgments of ADHD symptoms among Hispanic and White teachers. *Sch. Psychol. Q.* 20 268–287. 10.1521/scpq.2005.20.3.268

[B9] Di Martino A. Zuo X.-N. Kelly C. Grzadzinski R. Mennes M. Schvarcz A. (2013). Shared and distinct intrinsic functional network centrality in autism and attention-deficit/hyperactivity disorder. *Biol. Psychiatry* 74 623–632. 10.1016/j.biopsych.2013.02.011 23541632 PMC4508007

[B10] DuPaul G. Power T. Anastopoulos A. Reid R. (1998). *ADHD Rating Scale—IV: Checklists, Norms, and Clinical Interpretation.* New York, NY: Guilford Press.

[B11] Ehlers S. Gillberg C. Wing L. (1999). A screening questionnaire for Asperger syndrome and other high-functioning autism spectrum disorders in school age children. *J. Autism Dev. Disord.* 29 129–141. 10.1023/A:1023040610384 10382133

[B12] Faul F. Erdfelder E. Buchner A. Lang A.-G. (2009). Statistical power analyses using G^∗^Power 3.1: tests for correlation and regression analyses. *Behav. Res. Methods* 41 1149–1160. 10.3758/BRM.41.4.1149 19897823

[B13] Getahun D. Jacobsen S. J. Fassett M. J. Chen W. Demissie K. Rhoads G. G. (2013). Recent trends in childhood attention-deficit/hyperactivity disorder. *JAMA Pediatr.* 167 282–288. 10.1001/2013.jamapediatrics.401 23338799

[B14] Hall E. T. (1976). *Beyound Culture.* New York, NY: Anchor Books.

[B15] Howes D. H. Solomon R. L. (1951). Visual duration threshold as a function of word-probability. *J. Exp. Psychol.* 41 401–410. 10.1037/h0056020 14873866

[B16] Ishii K. Reyes J. A. Kitayama S. (2003). Spontaneous attention to word content versus emotional tone: differences among three cultures. *Psychol. Sci.* 14 39–46. 10.1111/1467-9280.01416 12564752

[B17] Kikuchi T. (2011). *Image of Undergraduate Students would-be Educator Toward Children with Developmental Disorders -Relation of Contact Experience and knowledge.* Available at: http://hdl.handle.net/2298/18552 [accessed March 31 2017].

[B18] Kitayama S. Markus H. R. Kurokawa M. (2000). Culture, emotion, and well-being: good feelings in Japan and the United states. *Cogn. Emot.* 14 93–124. 10.1080/026999300379003

[B19] Le Couteur A. Rutter M. Lord C. Rios P. Robertson S. Holdgrafer M. (1989). Autism diagnostic interview: a standardized investigator-based instrument. *J. Autism Dev. Disord.* 19 363–387. 10.1007/BF02212936 2793783

[B20] Liu Y. Li J. Zheng Q. Zaroff C. M. Hall B. J. Li X. (2016). Knowledge, attitudes, and perceptions of autism spectrum disorder in a stratified sampling of preschool teachers in China. *BMC Psychiatry* 16 142–153. 10.1186/s12888-016-0845-2 27177619 PMC4865992

[B21] Mann M. Mueller C. W. Ph D. Tao K. T. Humris E. Li B. L. (1992). Cross-cultural differences in rating hyperactive-disruptive behaviors in children. *Am. J. Psychiatry* 149 1539–1542. 10.1176/ajp.149.11.1539 1415822

[B22] Mason B. A. Gunersel A. B. Ney E. A. (2014). Cultural and ethnic bias in teacher ratings of behavior: a criterion-focused review. *Psychol. Sch.* 51 1017–1030. 10.1002/pits.21800

[B23] Matson J. L. Kozlowski A. M. (2011). The increasing prevalence of autism spectrum disorders. *Res. Autism Spectr. Disord.* 5 418–425. 10.1016/j.rasd.2010.06.004

[B24] Matson J. L. Matheis M. Burns C. O. Esposito G. Venuti P. Pisula E. (2017). Examining cross-cultural differences in autism spectrum disorder: a multinational comparison from Greece, Italy, Japan, Poland, and the United States. *Eur. Psychiatry* 42 70–76. 10.1016/j.eurpsy.2016.10.007 28212508

[B25] Matson J. L. Worley J. A. Fodstad J. C. Chung K.-M. Suh D. Jhin H. K. (2011). A multinational study examining the cross cultural differences in reported symptoms of autism spectrum disorders: Israel, South Korea, the United Kingdom, and the United States of America. *Res. Autism Spectr. Disord.* 5 1598–1604. 10.1016/j.rasd.2011.03.007

[B26] Mayou R. Hawton K. (1986). Psychiatric disorder in the general hospital. *Br. J. Psychiatry* 149 172–190. 10.1192/bjp.149.2.172 3535978

[B27] Miodovnik A. Harstad E. Sideridis G. Huntington N. (2015). Timing of the diagnosis of attention-deficit/hyperactivity disorder and Autism spectrum disorder. *Pediatrics* 136 e830–e837. 10.1542/peds.2015-1502 26371198

[B28] Monden Y. Dan I. Nagashima M. Dan H. Uga M. Ikeda T. (2015). Individual classification of ADHD children by right prefrontal hemodynamic responses during a go/no-go task as assessed by fNIRS. *NeuroImage* 9 1–12. 10.1016/j.nicl.2015.06.011 26266096 PMC4528046

[B29] Nicolson R. Castellanos F. X. (2000). Commentary: considerations on the pharmacotherapy of attention deficits and hyperactivity in children with autism and other pervasive developmental disorders. *J. Autism Dev. Disord.* 30 461–462. 10.1023/A:1005511809545 11098884

[B30] Nijmeijer J. S. Hoekstra P. J. Minderaa R. B. Buitelaar J. K. Altink M. E. Buschgens C. J. M. (2009). PDD symptoms in ADHD, an independent familial trait? *J. Abnorm. Child Psychol.* 37 443–453. 10.1007/s10802-008-9282-0 19051006

[B31] Perry R. (1998). Misdiagnosed ADD/ADHD; rediagnosed PDD. *J. Am. Acad. Child Adolesc. Psychiatry* 37 113–114. 10.1097/00004583-199801000-00024 9444907

[B32] R Core Team (2017). *R: A Language and Environment for Statistical Computing.* Available at: https://www.r-project.org/ [accessed July 31 2017].

[B33] Ravindran N. Myers B. J. (2012). Cultural influences on perceptions of health, illness, and disability: a review and focus on Autism. *J. Child Fam. Stud.* 21 311–319. 10.1007/s10826-011-9477-9

[B34] Ray D. T. (1998). *Japanese Culture and Communication: Critical Cultural Analysis.* Lanham, MD: University Press of America.

[B35] Reid R. DuPaul G. J. Power T. J. Anastopoulos A. D. Rogers-Adkinson D. Noll M. B. (1998). Assessing culturally different students for attention deficit hyperactivity disorder using behavior rating scales. *J. Abnorm. Child Psychol.* 26 187–198. 10.1023/A 9650625

[B36] Ruggero C. J. Zimmerman M. Chelminski I. Young D. (2010). Borderline personality disorder and the misdiagnosis of bipolar disorder. *J. Psychiatr. Res.* 44 405–408. 10.1016/j.jpsychires.2009.09.011 19889426 PMC2849890

[B37] Sun X. Allison C. (2010). A review of the prevalence of autism spectrum disorder in Asia. *Res. Autism Spectr. Disord.* 4 156–167. 10.1016/j.rasd.2009.10.003

[B38] Takahara M. Tsuda Y. (2012). Familiarity degree of the terms about developmental disability in general public. *Res. Bull. Naruto Univ. Educ.* 27 94–99.

[B39] The Administrative Evaluation Bureau (2017). *Hattatsu Shogaisha Shien ni Kansuru Gyosei Hyoka/Kanshi Kekka Hokokusho [Administrative Evaluation and Monitoring about Support for Persons with Developmental Disabilities].* Available at: http://www.soumu.go.jp/main_content/000458776.pdf [Accessed March 31 2017].

[B40] The National Institute of Special Needs Education Japan [NISE] (2015). Recent policy and status on special needs education in Japan recent data on education for children with disabilities in Japan. *NISE Bull.* 14 29–36.

[B41] Thomas R. Sanders S. Doust J. Beller E. Glasziou P. (2015). Prevalence of attention-deficit/hyperactivity disorder: a systematic review and meta-analysis. *Pediatrics* 135 e994–e1001. 10.1542/peds.2014-3482 25733754

[B42] Vázquez-Costa M. Costa-Alcaraz A. M. (2013). Premature diagnostic closure: an avoidable type of error. *Rev. Clin. Esp.* 213 158–162. 10.1016/j.rce.2012.05.012 22818221

[B43] Wang Q. Cavanagh P. Green M. (1994). Familiarity and pop-out in visual search. *Percept. Psychophys.* 56 495–500. 10.3758/BF03206946 7991347

[B44] Willcutt E. G. (2012). The prevalence of DSM-IV attention-deficit/hyperactivity disorder: a meta-analytic review. *Neurotherapeutics* 9 490–499. 10.1007/s13311-012-0135-8 22976615 PMC3441936

[B45] Wing L. Leekam S. R. Libby S. J. Gould J. Larcombe M. (2002). The diagnostic interview for social and communication disorders: background, inter-rater reliability and clinical use. *J. Child Psychol. Psychiatry Allied Discip.* 43 307–325. 10.1111/1469-7610.00023 11944874

[B46] Yoshida Y. Uchiyama T. (2004). The clinical necessity for assessing attention deficit/hyperactivity disorder (AD/HD) symptoms in children with high-functioning pervasive developmental disorder (PDD). *Eur. Child Adolesc. Psychiatry* 13 307–314. 10.1007/s00787-004-0391-1 15490278

[B47] Zhu C. Z. Zang Y. F. Cao Q. J. Yan C. G. He Y. Jiang T. Z. (2008). Fisher discriminative analysis of resting-state brain function for attention-deficit/hyperactivity disorder. *Neuroimage* 40 110–120. 10.1016/j.neuroimage.2007.11.029 18191584

