# Supplementary material for: Biases in Understanding Attention Deficit Hyperactivity Disorder and Autism Spectrum Disorder in Japan
Source: Front Psychol. 2018 Feb 28;9:244. doi: 10.3389/fpsyg.2018.00244 (PMC5836146; doi:10.3389/fpsyg.2018.00244)
Supplement: Supplementary file 1 [file Table_1.docx]

Supplementary Material

Biases in Understanding Attention Deficit Hyperactivity Disorder and Autism Spectrum Disorder in Japan

Mami Miyasaka^*^, Shogo Kajimura, Michio Nomura

***Correspondence:** Mami Miyasaka: miyasaka.mami.57e@kyoto-u.jp

Table S1

*Psychological Similarities between ADHD and ASD in Detail (N = 34)*

| Response category | *F* | % |
| --- | --- | --- |
| Overt characteristics |  |  |
| Difficulty understanding nonverbal information  and/or situations | 9 | 16.4 |
| Difficulty understanding others’ emotion | 6 |  |
| Difficulty understanding situations | 2 |  |
| Difficulty understanding essence | 1 |  |
| Issues with emotion | 6 | 10.9 |
| Difficulty in regulating emotion | 5 |  |
| Difficulty in expressing emotion | 1 |  |
| Issues involving cooperation with others | 5 | 9.1 |
| Lack of empathy | 1 |  |
| Difficulty understanding others’ perspectives | 1 |  |
| Interpersonal problems or maladaptation  in group settings | 1 |  |
| Lack of cooperation | 1 |  |
| Preference for keeping to oneself | 1 |  |
| Difficulty communicating | 4 | 7.3 |
| Communication difficulties | 2 |  |
| Difficulty understanding conversation | 1 |  |
| Difficulty understanding others’ words | 1 |  |
| Deviation of interest or concern | 4 | 7.3 |
| Narrowness of interest or concern | 2 |  |
| Expression of interest only in current activities | 1 |  |
| Rich knowledge of interests | 1 |  |
| Issues involving executive function | 3 | 5.5 |
| Issues involving attention control | 2 |  |
| Issues involving executive function | 1 |  |
| Poor flexibility | 3 | 5.5 |
| Difficulty paying attention to surroundings  during concentration | 2 |  |
| Strong persistence | 1 |  |
| Self-control difficulties | 3 | 5.5 |
| Difficulty controlling behavior | 2 |  |
| Self-control difficulties | 1 |  |
| Other overt characteristics | 11 | 20.0 |
| Characteristics related to five sense | 2 |  |
| Difficulty planning or preparing for the future | 2 |  |
| Failure to listen to others | 2 |  |
| Dislike of direction from others | 1 |  |
| Issues involving social skills | 1 |  |
| Similarity in appearance or characteristics | 1 |  |
| Thinking pattern | 1 |  |
| Visual perception differs from typical development | 1 |  |
| Secondary Issues |  |  |
| Decline in self-evaluation | 4 | 7.3 |
| Tendency toward decline in self-confidence  or self-esteem | 4 |  |
| Interventions |  |  |
| Effectiveness of environmental regulation | 3 | 5.5 |
| Effectiveness of environmental regulation | 3 |  |
| Total | 55 | 100 |
| *Note*. Multiple responses were valid; four missing responses. | | |
