# Supplementary material for: Biases in Understanding Attention Deficit Hyperactivity Disorder and Autism Spectrum Disorder in Japan
Source: Front Psychol. 2018 Feb 28;9:244. doi: 10.3389/fpsyg.2018.00244 (PMC5836146; doi:10.3389/fpsyg.2018.00244)
Supplement: Supplementary file 2 [file Table_2.docx]

Supplementary Material

Biases in Understanding Attention Deficit Hyperactivity Disorder and Autism Spectrum Disorder in Japan

Mami MIYASAKA^*^, Shogo KAJIMURA, Michio NOMURA

***Correspondence:** Mami MIYASAKA: miyasaka.mami.57e@kyoto-u.jp

Table S2

*Similarity and Difficulty Ratings (N = 47)*

|  | Similarity | |  | Difficulty | |
| --- | --- | --- | --- | --- | --- |
|  | *M* | *SD* |  | *M* | *SD* |
| Anxiety disorder | 3.09 | 1.30 |  | 3.38 | 1.21 |
| Autism spectrum disorder | 4.38 | 1.53 |  | 4.30 | 1.33 |
| Bipolar disorder | 4.28 | 1.23 |  | 4.13 | 1.35 |
| Conduct disorder | 4.96 | 1.61 |  | 4.57 | 1.51 |
| Depression | 2.96 | 1.43 |  | 3.34 | 1.26 |
| Disinhibited social engagement disorder | 5.06 | 1.54 |  | 4.96 | 1.44 |
| Intellectual disorder | 3.57 | 1.49 |  | 3.34 | 1.37 |
| Oppositional defiant disorder | 5.13 | 1.64 |  | 4.64 | 1.50 |
| Hyperactivity in typical development |  |  |  | 4.04 | 1.37 |
| Impulsivity in typical development |  |  |  | 4.00 | 1.30 |
| Inattention in typical development |  |  |  | 4.02 | 1.31 |
| *Note*. *M*, mean; *SD*, standard deviation. Similarity responses ranged from 1 (*extremely dissimilar*) to 7 (*extremely similar*); difficulty responses ranged from 1 (*very easy*) to 7 (*very difficult*). | | | | | |
