# Supplementary material for: Biases in Understanding Attention Deficit Hyperactivity Disorder and Autism Spectrum Disorder in Japan
Source: Front Psychol. 2018 Feb 28;9:244. doi: 10.3389/fpsyg.2018.00244 (PMC5836146; doi:10.3389/fpsyg.2018.00244)
Supplement: Supplementary file 3 [file Table_3.docx]

Supplementary Material

Biases in Understanding Attention Deficit Hyperactivity Disorder and Autism Spectrum Disorder in Japan

Mami MIYASAKA^*^, Shogo KAJIMURA, Michio NOMURA

***Correspondence:** Mami MIYASAKA: miyasaka.mami.57e@kyoto-u.jp

Table S3

*Results of Multiple Comparisons of Similarity and Difficulty Ratings via Wilcoxon’s Signed-Rank Test (N = 47)*

|  | ASD | | ODD | | DSED | |  |  |  |  |
| --- | --- | --- | --- | --- | --- | --- | --- | --- | --- | --- |
|  | *V* | *p* | *V* | *p* | *V* | *p* |  |  |  |  |
| **Similarities** |  |  |  |  |  |  |  |  |  |  |
| ASD |  |  | 48.5 | .054 | 100.5 | .211 |  |  |  |  |
| ODD | 357.5 | .054 |  |  | 140.0 | 1.000 |  |  |  |  |
| DSED | 395.5 | .211 | 113.0 | 1.000 |  |  |  |  |  |  |
| **Difficulties** |  |  |  |  |  |  |  |  |  |  |
| ASD |  |  | 151.0 | 1.000 | 101.0 | .422 |  |  |  |  |
| ODD | 284.0 | 1.000 |  |  | 76.0 | 1.000 |  |  |  |  |
| DSED | 395.0 | .422 | 200.0 | 1.000 |  |  |  |  |  |  |
|  | ANX | | BIP | | CD | | DEP | | ID | |
|  | *V* | *p* | *V* | *p* | *V* | *p* | *V* | *p* | *V* | *P* |
| **Similarities** |  |  |  |  |  |  |  |  |  |  |
| ASD | 546.0^**^ | <.001 | 261.5 | 1.000 | 71.0 | .254 | 514.5^**^ | <.001 | 444.5^*^ | .011 |
| ODD | 727.0^**^ | <.001 | 429.5^*^ | .035 | 40.5 | .693 | 691.5^**^ | <.001 | 604.5^**^ | <.001 |
| DSED | 678.0^**^ | <.001 | 460.5^*^ | .040 | 156.0 | 1.000 | 739.0^**^ | <.001 | 665.5^**^ | <.001 |
| **Difficulties** |  |  |  |  |  |  |  |  |  |  |
| ASD | 545.5^*^ | .032 | 346.5 | 1.000 | 202.0 | 1.000 | 408.0^**^ | .009 | 402.0^**^ | .002 |
| ODD | 504.0^**^ | .003 | 344.0 | 1.000 | 8.0 | 1.000 | 563.0^**^ | .002 | 513.5^**^ | .001 |
| DSED | 601.5^**^ | <.001 | 385.0^*^ | .019 | 240.0 | 1.000 | 580.5^**^ | <.001 | 723.0^**^ | <.001 |
|  | TDha | | TDim | | TDia | |  |  |  |  |
|  | *V* | *p* | *V* | *p* | *V* | *p* |  |  |  |  |
| **Difficulties** |  |  |  |  |  |  |  |  |  |  |
| ASD | 251.0 | 1.000 | 278.5 | 1.000 | 291.5 | 1.000 |  |  |  |  |
| ODD | 314.0 | .586 | 363.0 | .361 | 299.5 | .405 |  |  |  |  |
| DSED | 478.5^*^ | .025 | 503.5^**^ | .004 | 486.0^*^ | .015 |  |  |  |  |
| *Note.* ASD, autism spectrum disorder; ODD, oppositional defiant disorder; DSED, disinhibited social engagement disorder; ANX, anxiety disorder; BIP, bipolar disorder; CD, conduct disorder; DEP, depression; ID, intellectual disorder; TDha, hyperactivity in typical development; TDim, impulsivity in typical development; TDia, inattention in typical development. *p* values calculated via Bonferroni correction. **p ­*< .05; ***p* < .01 | | | | | | | | | | |
