# Supplementary material for: Biases in Understanding Attention Deficit Hyperactivity Disorder and Autism Spectrum Disorder in Japan
Source: Front Psychol. 2018 Feb 28;9:244. doi: 10.3389/fpsyg.2018.00244 (PMC5836146; doi:10.3389/fpsyg.2018.00244)
Supplement: Supplementary file 4 [file Data_Sheet_1.docx]

Supplementary Material

Biases in Understanding Attention Deficit Hyperactivity Disorder and Autism Spectrum Disorder in Japan

Mami MIYASAKA^*^, Shogo KAJIMURA, Michio NOMURA

***Correspondence:** Mami MIYASAKA: miyasaka.mami.57e@kyoto-u.jp

Supplement 1.

*Development of Assessment task*

Fictional cases were constructed based on DSM-5 criteria for autism spectrum disorder, oppositional defiant disorder, disinhibited social engagement disorder, and attention deficit hyperactivity disorder and limited to observable behavioral characteristics. The four cases were as follows (all stories consisted of 70 words, excluding periods or commas, in Japanese).

In a preliminary investigation, we asked six clinical psychologists who had been working in the field of child support for at least 2 years to assess each case. Participants read each sentence and selected the problem they considered the most probable diagnosis for 16 psychological problems observed in children. We excluded one participant who assessed three cases as pervasive developmental disorder, because this led to doubt regarding accuracy. Using the responses provided by five participants (four women, *M*age = 32.60, *SD* = 3.21 years, *M*exp = 5.4, *SD* = 2.2 years), we accepted responses that had been provided by at least four participants (>80%) for all four cases. The correct responses for the cases below (in ascending order) were autism spectrum disorder, oppositional defiant disorder, attention deficit hyperactivity disorder, and disinhibited social engagement disorder.

**Case 1: L, a boy in the fifth grade at elementary school O**

He panics when schedules are changed. Although he tries to talk about numerous things, his speech is so confused that we are just not on the same wavelength. Because he speaks without considering the situation or others’ feelings, he has few friends.

**Case 2: C, a boy in the fifth grade at elementary school K**

His dismissive attitude toward instructions from the teacher in class is striking. His stubborn attitude irritates adults, and he blames others for his own failures. Therefore, he is likely to cause trouble.

**Case 3: N, a boy in the fifth grade at elementary school R**

During class, he does not seem to understand instructions from the teacher, and his hands and feet are always moving. Furthermore, because he cuts in when classmates are playing, he is likely to cause trouble.

**Case 4: S, a boy in the fifth grade at elementary school Z**

While at school, he calls out to strangers (i.e., adults or younger or older children) of his own accord. However, classmates complained to the teacher about him, stating that they were distressed by his overfamiliar manner.
